# Short-term exposure to cyan light attenuates myopigenic effects of hyperopic defocus on ocular biometry in humans

**DOI:** 10.1038/s41598-026-35377-w

**Published:** 2026-01-09

**Authors:** Azfira Hussain, Eleonore Pic, Konogan Baranton, Pascale Lacan, Giovanni Diana, Nicola S Anstice, Aarti Gulyani, Montserrat Burgos, Ranjay Chakraborty

**Affiliations:** 1 Myopia and Visual Development Lab, College of Nursing and Health Sciences, Caring Futures Institute, Flinders University, Adelaide, South Australia Australia; 2 Neuroscience and Medical Research Centre, EssilorLuxottica, Paris, France; 3 Optometry Australia, Adelaide, South Australia Australia; 4 Caring Future Institute, College of Nursing and Health Sciences, Flinders University, Adelaide, South Australia Australia

**Keywords:** Myopia, Axial length, Choroidal thickness, Cyan light, Hyperopic defocus, Narrowband wavelength, Diseases, Medical research, Optics and photonics

## Abstract

This study investigated whether short-term exposure to cyan light can inhibit axial elongation and choroidal thinning induced by myopigenic hyperopic defocus in humans. Thirteen myopes (-3.73 ± 1.21 D) and fifteen emmetropes (-0.05 ± 0.11 D), aged of 24.07 ± 3.54 years, were exposed to cyan light (507 nm; total irradiance 3.06 W/m^2^) and broadband light (3.05 W/m^2^) using light-emitting glasses for two hours on separate days. Hyperopic defocus (-3D) was imposed on right eye using contact lenses (CL), while the fellow eye experienced no defocus. Ocular biometry, including axial length (AL) and subfoveal choroidal thickness (SFCT), was measured at baseline, after 60 and 120 min of light exposure, and 30 min post light offset. Three-way repeated measures ANOVA revealed that the effects of broadband and cyan light on AL (defocus × time × wavelength interaction, F _(3,162)_ = 0.37, *p* = 0.777) and SFCT (F _(3,162)_ = 0.35, *p* = 0.787) across all time points did not differ significantly between defocused and non-defocused eyes. However, exposure to cyan light significantly reduced axial elongation (mean difference in AL between cyan and broadband light: −0.011 ± 0.002 mm; Holm–Sidak post hoc test, *p* = 0.001) and increased SFCT (mean difference: +0.009 ± 0.002 mm; *p* < 0.001) at 120 min, compared with eyes exposed to the same degree of hyperopic defocus under broadband light. In non-defocused eyes, cyan light resulted in a significant increase in SFCT compared to broadband light at 120 min (mean difference, + 0.010 ± 0.001 mm, *p* < 0.001), while no significant difference was observed in AL. No significant effect of refractive error was observed in either eye (*p* > 0.05). Short-term exposure to cyan light can inhibit AL elongation and choroidal thinning induced by hyperopic defocus, warranting further investigations into its long-term potential for myopia control.

**Supplementary Information:**

The online version contains supplementary material available at 10.1038/s41598-026-35377-w.

## Introduction

The alarming prevalence of myopia poses a significant public health concern due to its progressive nature and potential for vision-threatening complications^1^, prompting extensive investigation into its underlying mechanisms. This has spurred increasing interest in preventive strategies, with ambient light exposure emerging as a key area of focus. Increased time outdoors has been shown to be associated with delayed myopia onset in children^2,3^. This protective effect of time outdoors has been attributed to both neurochemical factors, such as release of dopamine^4^, as well as optical factors, including reduced peripheral hyperopic defocus^5^, relaxed accommodation^6^, and increased spatial frequency content in outdoor environment^7^. One important factor could be the difference in the spectral composition of outdoor versus indoor light in influencing eye growth and refractive development^8^. The impact of the spectral composition of outdoor light on myopia may be attributed to longitudinal chromatic aberration (LCA), where shorter wavelength blue light is refracted more than longer wavelength red light, generating a chromatic cue for the sign of defocus^9^. Gawne and Norton proposed that emmetropisation compares the image contrast produced by short and long wavelength sensitive cones, enabling the eye to make compensatory adjustments that guide it toward emmetropia^10^. In addition, the changes in eye growth observed during outdoor exposure could be effects of specific wavelengths within the light spectrum^11^.

Experimental animal models have shown varying effects of exposure to different narrowband wavelengths on ocular growth and refractive development^12^. For instance, guinea pigs^13^, fish^14,15^ and chicks^16^ develop myopia when reared under middle or long wavelength (green or red) light and hyperopia when reared under short-wavelength (violet and blue) light^17–19^. In contrast, rhesus monkeys^20^ and tree shrews^21,22^ raised under narrowband long wavelength have been shown to develop hyperopia. These variations in findings may be attributed to species-specific differences or differences in light characteristics used in the studies. Despite these observations, the underlying mechanisms by which different wavelengths and spectral compositions of light influence ocular growth remain unclear.

Exposure to different narrowband wavelengths has been explored as a potential light-based intervention for myopia control in humans, specifically in paediatric populations, through studies evaluating repeated low-level red-light (RLRL) therapy and violet light-emitting eyeglasses^23^. Long-wavelength narrowband red light delivered via RLRL therapy has demonstrated high efficacy in slowing myopia progression in children, although recent studies have highlighted potential concerns regarding ocular safety^24^, including retinal photoreceptor damage and phototoxicity^25–27^. In contrast, violet light eyewear has shown only modest effects in reducing myopia progression and axial elongation in children^28^. Consequently, identifying a safe and effective light-based intervention for myopia control remains an area of considerable interest^29–34^. In recent studies, whilst short-term exposure (60 min) to long-wavelength light (620–630 nm; bandwidth: 20–35 nm) delivered via light-emitting diode (LEDs) resulted in axial length (AL) elongation and choroidal thinning,^29,31^ exposure to short-wavelength blue light (454–456 nm; bandwidth: 20–25 nm)^29,31^ and middle-wavelength cyan light (507 nm; bandwidth 32 nm) resulted in significant AL shortening and choroidal thickening in young adults, with cyan light eliciting a stronger effect than blue light^30^. Similarly, Read et al. investigated the effects cyan light (500 nm) in young adults and found that daily morning exposure for 30 min over one week resulted in significant choroidal thickening^35^. These changes are hypothesised to be mediated by melanopsin retinal ganglion cells (mRGCs) and rod pathways^30^. It is important to note that these short-term changes in AL do not necessarily represent true structural alterations of the eyeball or longer-term changes in eye growth^36^. Instead, they represent small, acute changes in the retinal pigment epithelium (RPE) plane, resulting from dynamic changes in choroidal thickness^37^. Such rapid choroidal adjustments can transiently influence AL measurements, which is measured from the anterior cornea to the RPE via different optical biometers.

Previous research on animals demonstrated that eye growth is vision dependent and compensatory ocular changes occur in response to spectacle lens defocus, leading to predictable changes in eye growth and refractive development^38,39^. In human studies, brief (60–120 min) exposure to hyperopic defocus (negative lens) results in choroidal thinning and increase in AL^40,41^. Similar ocular changes have been observed in response to accommodation, which leads to axial elongation and thinning of choroid^42^. In contrast, short-term exposure to myopic defocus induces choroidal thickening and a reduction in AL. The changes in ocular biometry in response to optical defocus are typically of smaller magnitude, in order of ~ 10–20 μm^41^.

Exposure to bright light has been shown to mitigate the effects of imposed hyperopic defocus in animal models^43,44^. Similar protective effects of outdoor activity under bright light against near work (and associated hyperopic defocus) have been reported in children^45^. Furthermore, exposure to short-wavelength light (violet light, 380 nm and blue light, 470 nm) has been shown to inhibit the ocular effects of lens-induced hyperopic defocus in guinea pigs^46,47^, mice^48^, and even humans^31^. Taken together, these findings raise the question of whether cyan light might similarly modulate or inhibit hyperopic defocus–induced changes in ocular biometry in humans - an interaction that remains unexplored. Therefore, this study aimed to determine whether short-term (120 min) exposure to cyan light can inhibit axial elongation and choroidal thinning induced by ‘myopigenic’ hyperopic retinal defocus in young adult human subjects. We hypothesised that short-term exposure to cyan light would counteract the AL elongation and choroidal thinning induced by hyperopic defocus in human subjects.

## Results

### Within-session repeatability measurement

Supplementary Table 1 shows the mean within-subject standard deviation (SD) and coefficient of variation (COV) for the repeated measures collected at each measurement session across the two measurement days to illustrate the overall within-subject variability in the measurements. The within-session variability was small for AL (mean within-session SD and COV, 0.009 mm and 0.04%, respectively), but slightly greater for the subfoveal choroidal thickness (SFCT, 0.011 mm and 3.74%, Supplementary Table 1). Other ocular parameters exhibited small to moderate variability with within-subject SD ranging from 0.005 to 0.028 mm and COV ranging from 0.49% − 0.77% (Supplementary Table 1).

### Changes in axial length

**Fig. 1:**
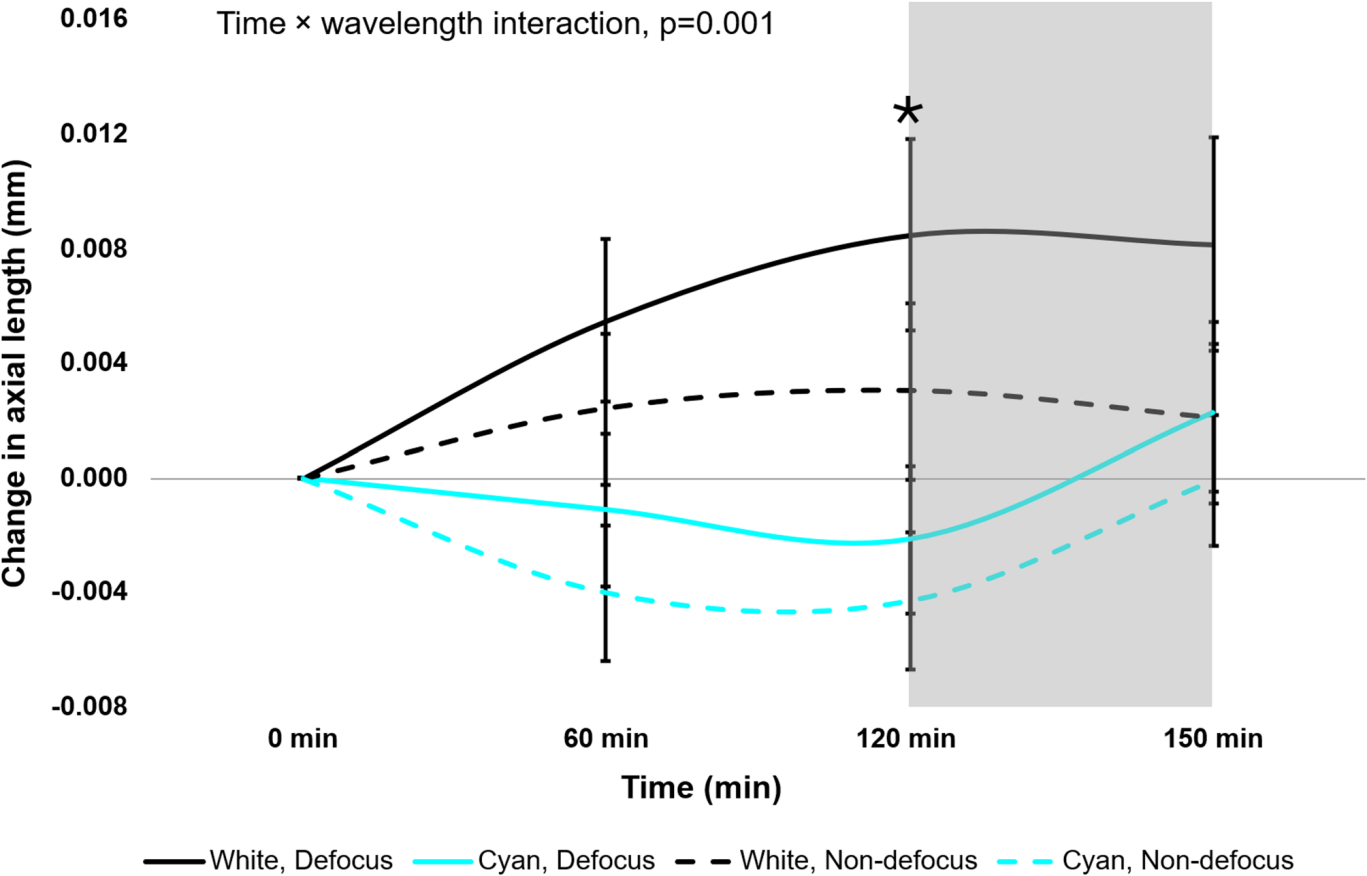
Changes in axial length for all participants (*n* = 28) to broadband and narrowband cyan lighting. Significant differences between the two light conditions in the defocused eye are indicated by black asterisks (*). Grey shaded area represents the recovery period.

The change in AL after exposure to broadband and cyan light in defocused and non-defocused eyes are shown in Fig. 1; Table 1. Three- way repeated-measures ANOVA showed that the effects of broadband and cyan light on AL across all time points did not differ significantly between the defocused and non-defocused eyes (defocus × time × wavelength interaction, F _(3,162)_ = 0.37, *p* = 0.777).

**Table 1:** Summary of overall mean change (SEM) in axial length (AL), central corneal thickness (CCT), corneal curvature (CC), anterior chamber depth (ACD), lens thickness (LT), vitreous chamber depth (VCD) and subfoveal choroidal thickness (SFCT) at 60 and 120 min of light exposure and after 30 min of light offset (recovery measured at 150 min) for each of the two light conditions (broadband light, and cyan light) in both eyes (defocused and non-defocused eyes), along with p-values from three-way repeated measures ANOVA illustrating the main effects of time, wavelength, defocus and their interactions.

Measured variables	Defocus	Wavelengths	Mean change at 60 min of light exposure (SEM; mm)	Mean change at 120 min of light exposure (SEM; mm)	Mean change after 30 min of offset (recovery measurement at 150 min; SEM; mm)	*p* (Time)	*p* (Wavelength)	*p* (Defocus)	*p* (Defocus × Time)	*p* (Defocus × Wavelength)	*p* (Time × Wavelength )	*p* (Time × Wavelength × Defocus )
AL	Defocused	Broadband light	+ 0.006 (0.003)	+ 0.009 (0.003)	+ 0.008 (0.004)	0.141	**0.004** ^*^	0.187	0.426	0.579	**0.001** ^*^	0.777
		Cyan light	-0.001 (0.003)	-0.002 (0.002)	+ 0.002 (0.003)							
	Non-defocused	Broadband light	+ 0.002 (0.003)	+ 0.003 (0.003)	+ 0.002 (0.003)							
		Cyan light	-0.004 (0.002)	-0.004 (0.002)	+ 0.000 (0.002)							
CC^a^	Defocused	Broadband light	-0.075 (0.035)	-0.074 (0.045)	-0.018 (0.041)	0.197^*^	0.883	**0.046** ^*^	0.088	0.575	0.998	0.803
		Cyan light	-0.051 (0.029)	-0.066 (0.185)	-0.017 (0.144)							
	Non-defocused	Broadband light	+ 0.031 (0.027)	+ 0.030 (0.033)	+ 0.026 (0.027)							
		Cyan light	-0.004 (0.047)	+ 0.019 (0.034)	+ 0.016 (0.030)							
CCT	Defocused	Broadband light	-0.019 (0.019)	-0.023 (0.021)	-0.004 (0.001)	0.272	0.671	0.456	0.715	0.158	0.815	0.193
		Cyan light	+ 0.006 (0.005)	+ 0.005 (0.005)	+ 0.000 (0.003)							
	Non-defocused	Broadband light	-0.001 (0.001)	-0.003 (0.001)	-0.003 (0.001)							
		Cyan light	-0.039 (0.036)	-0.036 (0.036)	-0.037 (0.036)							
ACD	Defocused	Broadband light	+ 0.009 (0.009)	+ 0.013 (0.012)	+ 0.027 (0.010)	**0.002** ^*^	0.208	0.638	0.332	0.814	0.380	0.310
		Cyan light	+ 0.000 (0.016)	-0.001 (0.016)	+ 0.007 (0.013)							
	Non-defocused	Broadband light	+ 0.007 (0.009)	+ 0.023 (0.012)	+ 0.033 (0.010)							
		Cyan light	-0.005 (0.012)	-0.008 (0.014)	+ 0.047 (0.031)							
LT	Defocused	Broadband light	+ 0.060 (0.055)	+ 0.025 (0.070)	+ 0.040 (0.052)	0.325	0.714	0.993	0.945	0.223	0.438	0.278
		Cyan light	-0.015 (0.040)	+ 0.029 (0.014)	+ 0.012 (0.012)							
	Non-defocused	Broadband light	+ 0.008 (0.009)	-0.005 (0.011)	-0.018 (0.007)							
		Cyan light	+ 0.056 (0.049)	+ 0.065 (0.050)	+ 0.046 (0.052)							
VCD	Defocused	Broadband light	-0.061 (0.055)	+ 0.010 (0.081)	-0.470 (0.477)	0.221	0.923	0.254	0.579	0.734	0.643	0.174
		Cyan light	-0.412 (0.303)	-0.145 (0.114)	-0.137 (0.113)							
	Non-defocused	Broadband light	-0.193 (0.180)	-0.012 (0.010)	-0.010 (0.009)							
		Cyan light	-0.023 (0.063)	-0.032 (0.066)	-0.064 (0.073)							
SFCT	Defocused	Broadband light	-0.001 (0.002)	-0.003 (0.002)	-0.002 (0.002)	**0.011** ^*^	**0.001** ^*^	0.845	0.974	0.734	**< 0.001** ^*^	0.787
		Cyan light	+ 0.002 (0.001)	+ 0.006 (0.002)	0.000 (0.001)							
	Non-defocused	Broadband light	-0.002 (0.002)	-0.004 (0.002)	-0.003 (0.001)							
		Cyan light	+ 0.002 (0.002)	+ 0.007 (0.002)	-0.001 (0.002)							

^a^The change in CC shown in dioptres (D).

*Significant p values (< 0.05) are highlighted in bold.

However, the effect of cyan light on AL was significantly different from that of broadband light over time for both defocused and non-defocused eyes (time × wavelength interaction, F _(3,162)_ = 5.46, *p* = 0.001). Post-hoc analysis showed that in defocused eye, cyan light caused a significant reduction in AL compared to broadband light at 120 (-0.011 ± 0.002 mm; Holm-Sidak post-hoc test, *p* < 0.001) but not at 60 min (-0.007 ± 0.003 mm, *p* = 0.130). In the non-defocused eye, no significant difference in AL was observed between broadband and cyan light at either 60 min (-0.006 ± 0.002 mm, Holm-Sidak post-hoc test, *p* = 0.160) or 120 min (-0.007 ± 0.002 mm, *p* = 0.052) (Fig. 1). The changes in AL observed under both broadband and cyan light conditions were transient in both defocused and non-defocused eye, returning to baseline within 30 min of light offset (*p* > 0.05), as shown in Table 1.

### Changes in choroidal thickness

**Fig. 2:**
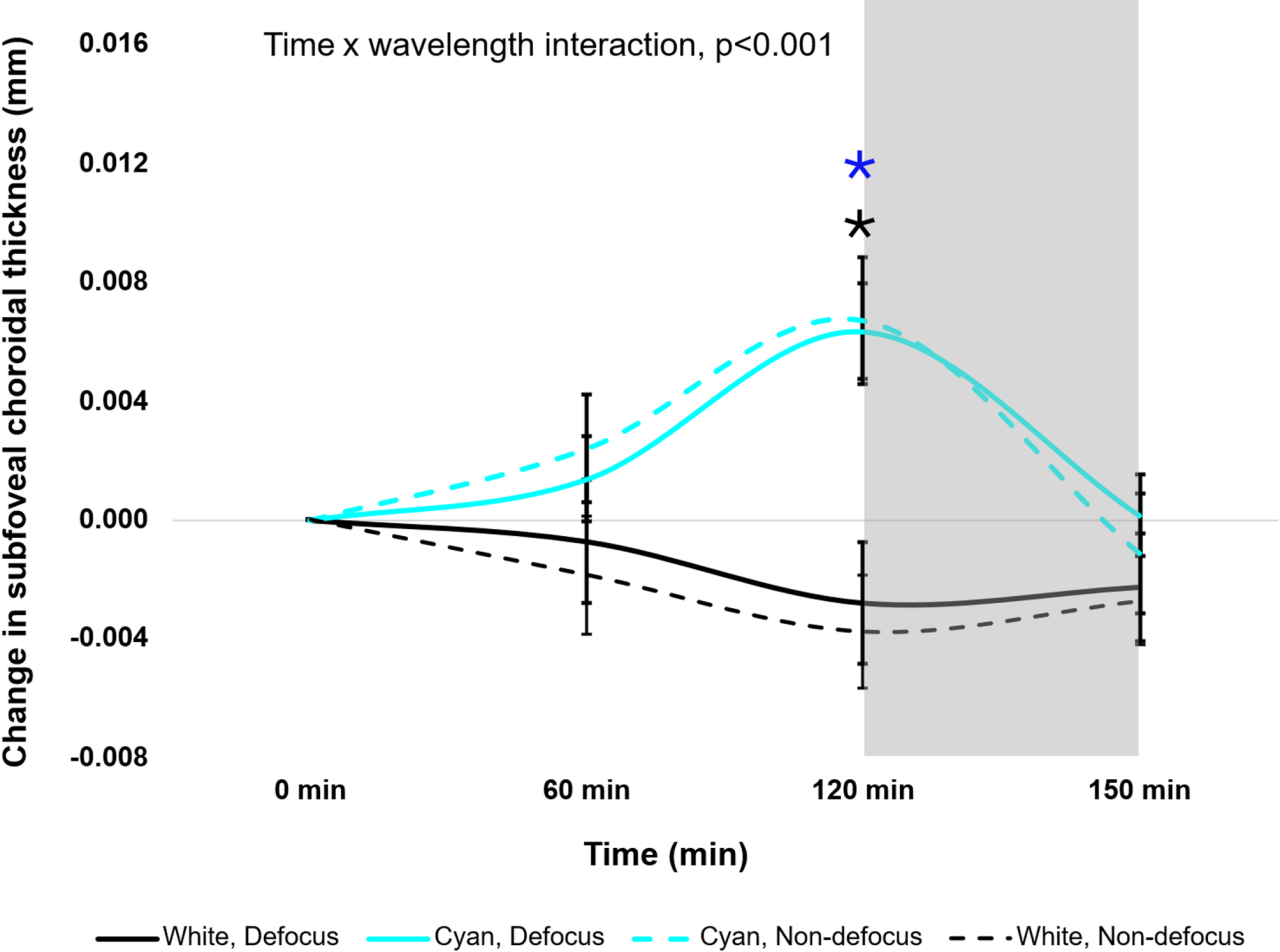
Changes in subfoveal choroidal thickness for all participants (*n* = 28) to broadband and narrowband cyan lighting. Significant differences between the two light conditions in the defocused eye are indicated by black asterisks and the non-defocused eye by blue asterisks. Grey shaded area represents the recovery period.

The change in SFCT associated with exposure to broadband and cyan light in defocused and non-defocused eyes are shown in Fig. 2; Table 1. Three-way repeated measures ANOVA showed that the effects of broadband and cyan light on SFCT across all time points was not significantly different between the defocused and non-defocused eyes (defocus × time × wavelength interaction, F _(3,162)_ = 0.35, *p* = 0.787).

However, the effect of cyan light on SFCT was significantly different from that of broadband light over time (time × wavelength interaction, F _(3,162)_ = 15.18, *p* < 0.001). Post-hoc analysis showed that in the defocused eye, exposure to cyan light resulted in a significant increase of + 0.009 ± 0.002 mm in SFCT at 120 min, compared to eyes subjected to the same degree of defocus under broadband light (Holm-Sidak post-hoc test, *p* < 0.001, Fig. 2). Similarly in the non-defocused eye, cyan light induced a significant increase in SFCT of + 0.010 ± 0.001 mm (*p* < 0.001) at 120 min compared with broadband light (Fig. 2). The changes in SFCT observed under the two light conditions were transient in both defocused and non-defocused light (*p* > 0.05), returning to baseline within 30 min of light offset, as shown in Table 1.

### Effect of refractive error

**Fig. 3:**
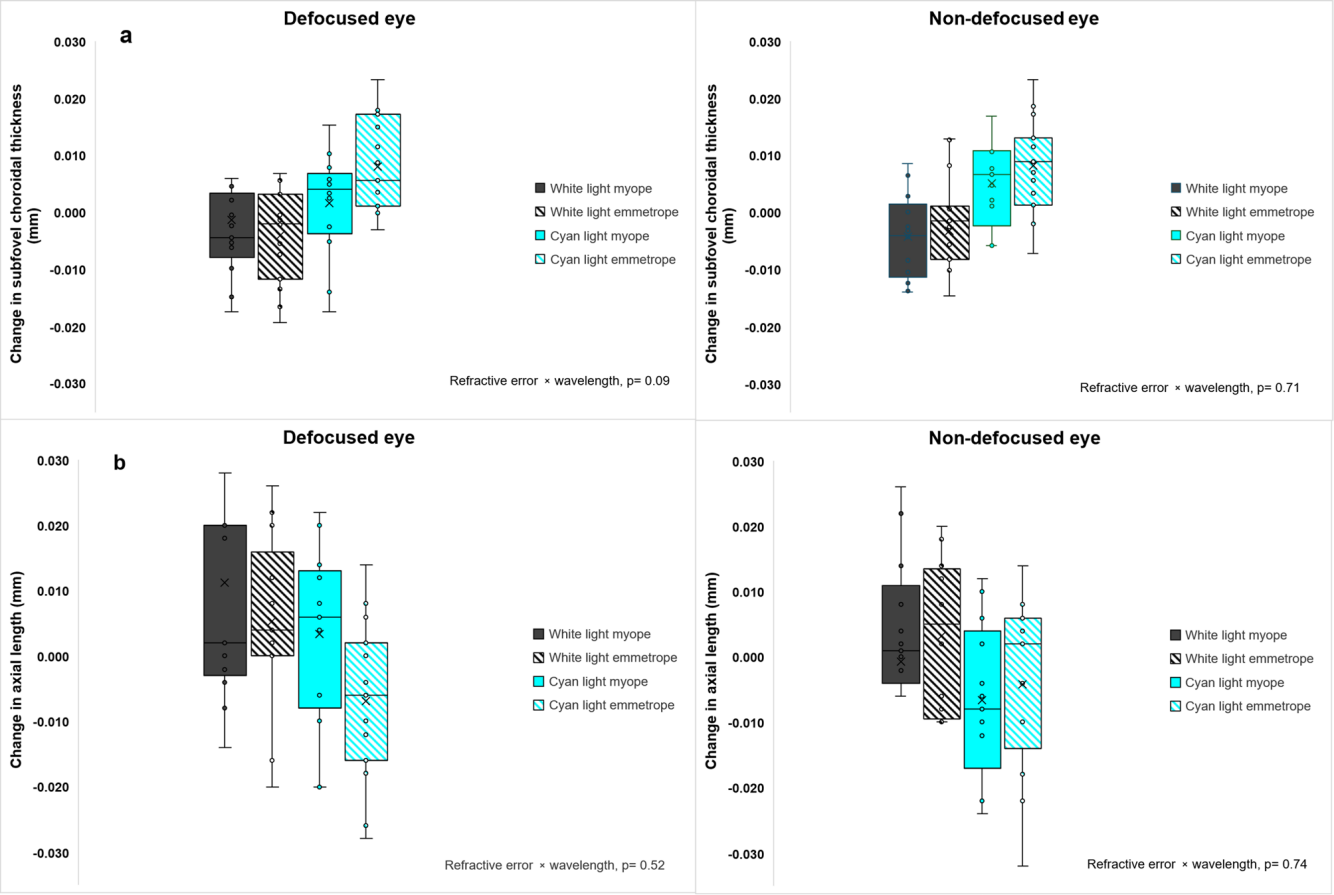
Axial length and subfoveal choroidal thickness changes to broadband and narrowband cyan lighting across the two refractive error groups. Top rows (**a**) illustrate the changes in subfoveal choroidal thickness for emmetropes (*n* = 15) and myopes (*n* = 13) at 120 min of light exposure. Bottom rows (**b**) illustrate the changes in axial length for emmetropes and myopes at 120 min of light exposure.

As shown in Figs. 1 and 2, the largest changes in AL and SFCT under cyan light occurred at 120 min in both defocused and non-defocused eyes. Figure 3 shows the 120-minute change in AL and SFCT under broadband and cyan light for defocused and non-defocused eyes across the two refractive groups. There was no significant interaction between defocus, wavelength, and refractive errors at 120 min for AL changes (F _(1,104)_ = 0.05, *p* = 0.818) or SFCT (F _(1,104)_ = 0.29, *p* = 0.592). Similarly, after 30 min of light offset (i.e., 150 min), no significant effect of refractive error was observed for AL (*p* = 0.575) or SFCT (*p* = 0.393) changes.

### Other ocular biometry parameters

The changes in ocular biometry parameters in response to different wavelengths for both defocused and non-defocused eyes are shown in Table 1. Introduction of defocus under both light conditions resulted in a significant decrease in corneal curvature (CC) (repeated measures ANOVA: main effect of defocus, F _(1,54)_ = 4.16, *p* = 0.046), which may have resulted from short-term CL wear. However, no significant CC changes were observed in the non-defocused eye (Table 1). Also, there was no significant effect of time or wavelength for CC change. Anterior chamber depth (ACD) varied across the measurement timepoints in both defocused and non-defocused eyes within each light condition (main effect of time, F _(3,162)_ = 5.39, *p* = 0.002). However, no significant interactions were noted between time × wavelength (F (_3,162)_ = 1.03, *p* = 0.380) or between defocus × time × wavelength (F _(3,162)_ = 1.20, *p* = 0.310).

### Accommodative response

**Fig. 4:**
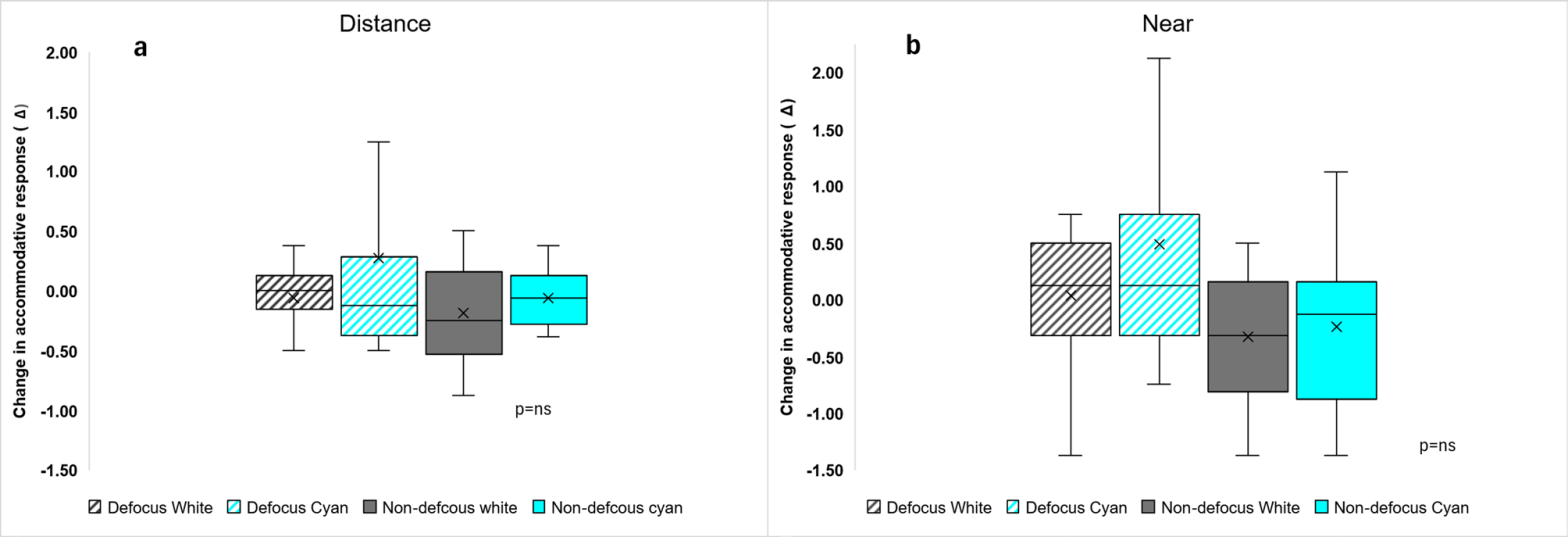
Mean objective refraction (*n* = 14) for broadband and cyan light in the defocused and non-defocused eyes. Left image (a) illustrates the accommodative response during binocular fixation at distance (4 m). Right image (b) illustrates the accommodative response during binocular fixation at near (30 cm).

There were no significant differences in the accommodative responses between the defocused and non-defocused eyes for either light condition at distance (defocus × wavelength interaction, F _(1,55)_ = 0.36, *p* = 0.553) and near (defocus × wavelength interaction, F _(1,55)_ = 0.06, *p* = 0.805) (Fig. 4).

## Discussion

The present study demonstrates that a brief exposure (120 min) to 507 nm cyan light induces significant inhibitory changes in ocular biometry in young adults. Specifically, cyan light attenuated the effects of monocular lens-induced hyperopic defocus by reducing AL elongation and choroidal thinning, compared to broadband light exposure.

In this study, the ocular biometry changes observed in the defocused eye under cyan light occurred in the opposite direction to the changes induced by broadband light. Specifically, compared to broadband light, exposure to cyan light for 120 min induced an AL reduction of 0.011 mm and a choroidal thickening of 0.009 mm. Similarly, a recent study found that 60 min of exposure to blue light (455 nm) inhibited the effects of lens-induced hyperopic defocus in young adults, leading to a reduction in AL and an increase in choroidal thickness compared to exposure to red (610 nm) and green (525 nm) light^31^. In contrast, a recent study in tree shrews found that rearing under narrowband cyan light (505 nm), both independently and in combination with monocular − 5 D lens wear, induced myopia and axial elongation^49^. This discrepancy may be attributed to interspecies differences in retinal structure and the dichromatic visual system of tree shrews^50,51^. However, the exact mechanisms underlying these ocular changes with cyan light exposure remains unclear and warrant further research.

Notably, the effects of cyan light on AL and SFCT in the non-defocused eye were generally similar to those observed in the defocused eye, suggesting that the inhibitory effect of cyan light under natural viewing conditions persists even in the presence of hyperopic defocus, in line with our hypothesis. While the mean reduction in AL in the non-defocused eye with cyan light was not statistically significant, cyan light exposure resulted in a significant increase in SFCT in both non-defocused and defocused eyes. These findings of reduced AL and increased SFCT following cyan light exposure are consistent with previous human studies^30,35^. Chakraborty et al. demonstrated that a 120 min exposure to narrowband cyan light (507 nm) led to significant AL reduction and choroidal thickening in both young adults and children using light emitting glasses^30^. Likewise, Read et al. that found that daily morning exposure to 500 nm cyan light for 30 min for one week resulted in significant thickening of the choroid^35^. Furthermore, exposure to cyan light has also been showed to be protective against axial myopia in zebrafish^52^.

It is noteworthy that although the effect of defocus on ocular biometry under broadband light was small and statistically insignificant, we still observed a significant difference between cyan and broadband light at 120 min. This difference would likely have been even greater if the defocused eye had exhibited more pronounced choroidal thinning and axial elongation in response to defocus. The relatively weaker effect of defocus observed under broadband light may be attributable to the characteristics of the light source used in this study. The broadband light (colour temperature of 6500 K) used in this study approximated a flatter spectrum similar to a D65 illuminant (a standard light source that represents average bright daylight defined by the International Commission on Illumination [CIE], https://cie.co.at/). It is possible that exposure to hyperopic defocus under a different spectrum of broadband light may have exerted greater changes in ocular biometry. We have previously shown that the spectral profile of broadband light can considerably impact ocular biometry responses in human subjects^30^.

In our study, we found no significant effect of refractive error on ocular biometry changes induced by cyan light in young adult participants. These findings were not consistent with our previous study, which reported a significant and sustained effect of cyan light on AL compared to broadband light in myopic (not emmetropic) participants^30^. This discrepancy may be attributed to several factors. First, a difference in baseline myopic refraction (current study: -3.73 ± 1.21 D vs. previous study: -3.03 ± 1.97 D) may have partly contributed to the observed disparity. Previous studies have shown that for every 1.00 D increase in myopia, choroidal thickness decreases by ~ 11–13 μm^53,54^. In the present study, the mean increase in choroidal thickness with cyan light was 7 μm in the non-defocused eye and 6 μm in the defocused eye; therefore, a 0.70 D difference in myopic refraction could account for ~ 50–60% of the difference in choroidal thickness between the two studies. Secondly, the analysis of refractive error was exploratory rather than a predefined outcome, and the sample size within each refractive error group was insufficient to reliably detect such differences. Future studies with larger sample sizes are warranted to better understand the role of refractive error in mediating ocular responses to cyan light in human subjects.

In humans, exposure to different wavelengths has been shown to affect accommodative response of the eye^55^, which in turn, can influence ocular biometry^42^. For example, it was shown that human subjects needed to exert less accommodation when they read under short-wavelength compared to long wavelength^55,56^. Furthermore, although the viewing distance was far, the introduction of optical defocus may also alter the accommodative behavior and defocus experienced by the contralateral eye^57,58^. In this study, no significant differences in accommodative responses were observed between the defocused and non-defocused eyes under any lighting condition, suggesting that neither optical defocus nor cyan light had a significant effect on accommodative function of the eye. These findings indicate that the effects of cyan light on ocular biometry are likely mediated by wavelength-dependent changes in retinal and choroidal signalling, rather than by changes in accommodation. This is further confirmed by small and insignificant changes in lens thickness (LT) and ACD under both light conditions. However, as measurements of accommodative response were taken only at a single time point (60 min), it does not preclude the possibility of small accommodative changes during the experiment which were not captured in this study.

Strengths of the study include the of investigating the effects of middle -wavelength 507 nm cyan light on ocular biometry in the context of optically induced hyperopic retinal defocus, contributing to the emerging evidence of its inhibitory impact on AL and choroidal thickness. However, this study has a few limitations. First, it was conducted on a modest sample size (*n* = 28) with smaller number of myopic and emmetropic participants. Future studies with larger sample sizes are required to elucidate refractive group differences between cyan and broadband light. Second, the study included only low- to moderate-myopic young adults, limiting the generalisability of the results to other age groups and a broader range of myopic refractive errors. Finally, the study only investigated the effects of negative lens-induced hyperopic defocus; future experiment incorporating positive lens-induced myopic defocus or employing filters/diffusers to simulate visual deprivation may further provide a more detailed understanding of the role of cyan light in myopia and refractive development.

In conclusion, short-term cyan light exposure can prevent AL elongation and choroidal thinning induced by ‘myopigenic’ hyperopic defocus, mirroring its effects under natural viewing conditions. This study contributes to the growing body of evidence supporting the potential inhibitory effects of cyan light on AL and choroidal thickness in humans. However, the study only demonstrates short-term effects of cyan light in ocular biometry and how these changes may affect longer-term changes in ocular growth remain unknown. Further research is needed to determine the long-term implications of cyan light exposure on myopia development and progression in humans.

## Methods

### Study participants

Twenty-eight participants (20 females) with a mean ± standard deviation, age of 24.07 ± 3.54 years were recruited to participate in the study. The study was approved by the Flinders University Human Research Ethics Committee (Flinders HREC, ID: 6748) and adhered to the principles of the Declaration of Helsinki. Participants were provided with detailed information about the study procedures, and written informed consent was obtained prior to participation. All participants provided consent for the publication of identifying images in online open-access publications. Prior to enrolment, all participants underwent a comprehensive eye examination and completed a brief General Health and Medical History questionnaire to screen for ocular health, general health conditions, sleep/circadian disorders, and medications that could potentially affect choroidal changes. The comprehensive eye examination included participants’ ocular and systemic history, assessments of visual acuity and refraction (objective and subjective), binocular vision and ocular motility testing, pupil assessment, slit lamp examination, colour vision testing and fundus examination (undilated). The study recruited participants with either myopia with a mean spherical equivalent refraction [*n* = 13, (SER) of -3.73 ± 1.21 D] or emmetropia (*n* = 15, mean SER, -0.05 ± 0.11 D). All participants, both myopes and emmetropes, had best-corrected visual acuity of 0.00 logMAR or better. The refractive error was measured using an autorefractor, followed by subjective refinement to obtain best-corrected visual acuity. Participants with previous history of ocular injury, surgery, or laser treatment; known systemic disease, seizures, or excessive sensitivity to light were excluded from the study.

### Experimental set up

**Fig. 5:**
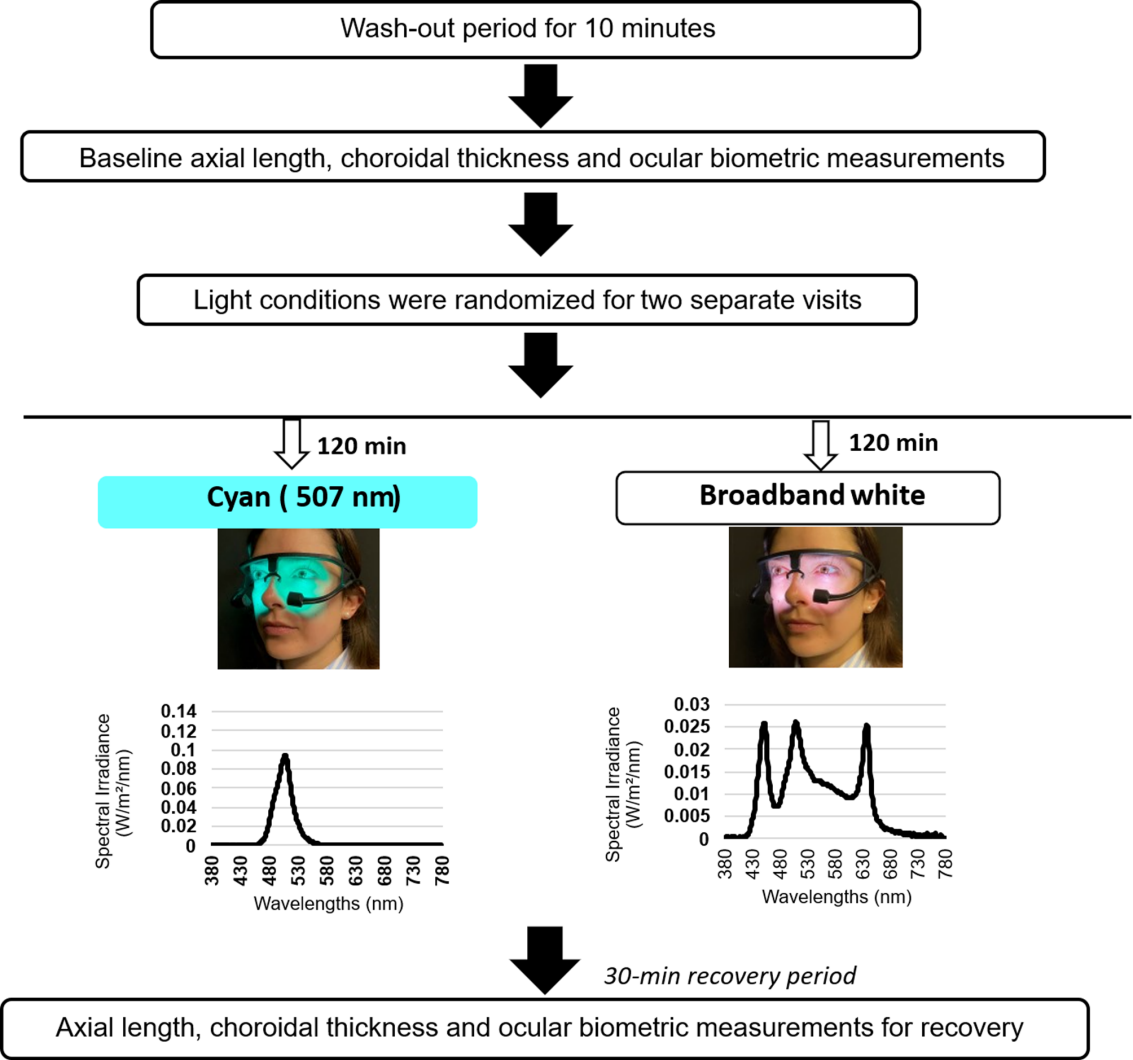
Flow diagram of experimental procedures including the setup and spectral profiles for cyan and broadband light conditions.

The detailed experimental set up is illustrated in Fig. 5. To evaluate the effect of cyan light exposure on AL and SFCT in response to hyperopic defocus, each participant was exposed to narrowband cyan light (peak wavelength: 507 nm; total irradiance: 3.06 W/m^2^; half-maximum band width: 32 nm) and broadband light (total irradiance: 3.05 W/m^2^) at eye level using light-emitting glasses for 120 min on two separate experimental days. The broadband light was generated using white, red and cyan LEDs to approximate a flatter spectrum across the visible range similar to the D65 illuminant. The cyan peak in the broadband light had an irradiance of 0.76 W/m^2^, which was approximately four times lesser than that of the narrowband cyan light (3.06 W/m^2^).

Following the light exposures, a series of ocular biometry measurements were obtained at baseline (before light exposure), at 60 and 120 min of light exposure, and at 30 minutes after light offset (150 min) to evaluate the recovery of light induced ocular changes in each eye. Irradiance levels at the corneal plane were assessed using a spectrophotometer (AvaSpec-ULS2048XL-USB2, Avantes, avantes.com/). The light-emitting glasses were designed from 3D-printed Nylon 12 polymer and incorporated two multi-color LED emitters (6 × 6 mm^2^), each covered with white diffusers (LuxiGen^®^, LZ7-04M2PD, Osram, osram.com/cb/). Each emitter contained seven closely packed LEDs within a low thermal resistance package, enclosed by an integrated glass window. As previously described^30^, these emitters were positioned at the lower portion of the plastic frame and projected light on the eye from a distance of ~ 4 cm. The desired wavelengths were controlled via a Java-based android smartphone application connected to the device through Bluetooth, with power supplied by a USB power bank. The device has been metrologically characterized by the LNE (Laboratoire National de Métrologie et d’Essais) to ensure that it is compliant with the photobiological safety standards (NF EN 62471, December 2008; https://piseo.fr/en/test-lab/photobiological-risk-evaluation-en62471/).

During the two experimental days, participants watched a movie of their choice of the same genre on a 1.12 m × 0.65 m television located 4 m away, corresponding to a field of view of 15.64° × 9.23°. Television was set to low brightness (26.4 lx measured at 1 m distance using lux meter), with all colour channels muted (i.e., grayscale) to prevent interference from other light sources and wavelengths on ocular biometry and accommodation^29,55^.

To induce hyperopic retinal defocus, a key stimulus for myopia development^59,60^, optical induction was used in this study. In experimental settings, hyperopic defocus can be induced either optically (using spectacles or CLs) or through near viewing of a target. Optical induction was preferred here as it offers greater experimental control minimising intrinsic variability in accommodative behaviour, whereas near viewing may introduce subtle changes in accommodative response due to small variations in viewing distance and gaze behaviour.

### Experimental protocol

On each day, before the baseline measurement (0 min), participants watched the television in dark for a period of 10 min (sitting and watching a television at 4 m) to wash out any residual effects of previous visual activities on measurements^30^. Following the 10 min wash out period, baseline measurements of AL and SFCT was performed. Participants were then fitted with a -3.00 D Proclear^®^ 1-day daily disposable contact lens (CL, CooperVision, Pleasanton, CA) in the right eye to induce hyperopic defocus, while the left eye received a plano (zero-powered) CL. The CLs have a mild blue visibility tint, which does not affect light transmission across the visible spectrum^61^. For myopic participants, the defocus was combined with the refractive correction into the CL power for the right eye, whereas the left eye received only the habitual refractive correction. The participants were given 5 min (or longer if requested) of adaptation time with CLs and asked to wear the light-emitting device and continue to watch television in a dimly lighted room (< 10 lx) for the remaining duration of the experiment. At each subsequent measurement session, participants were asked to remove the light-emitting glasses, after which the investigator removed the CLs immediately before the ocular biometry measurements. The CLs were kept safely in a CL case with solution and were gently reinserted on the eye at the end of the measurement. All the measurements were performed between 9:00 and 12:00 h to avoid the influence of diurnal variation on the ocular measurements^62^. The two lighting conditions were randomised to control for systematic bias, and all participants completed both experimental sessions within 10 days. Participants were instructed to refrain from consuming caffeinated beverages^63,64^ and performing high intensity exercises^65^ on the day of the experiment, as these have been shown to affect choroidal thickness.

To determine AL and other ocular biometry parameters- including CC, central corneal thickness (CCT), ACD, LT and vitreous chamber depth (VCD), a non-contact optical biometer (Lenstar LS 900; Haag-Streit AG, Koeniz, Switzerland) was used for the measurement. Measurements were performed first in the defocused eye, followed by the non-defocused eye, for all the participants. When a scan was identified by the instrument as having low reliability, it was repeated until it met the Lenstar’s internal quality criteria. The SFCT was measured using Cirrus HD spectral domain optical coherence tomography (SD-OCT; Cirrus HD-OCT 5000, Carl Zeiss Meditech Inc.). At each measurement session, the scanning protocol included two 6 mm high-definition 5-line raster scans (signal strength ≥ 8) from each eye using enhanced depth imaging. A single line passing through the fovea, consisting of 5 B scans, with 1024 A scans per B scan, was used for analysis^30^.

Previous studies have shown significant changes in AL and ChT associated with accommodation in human subjects^42^. A subset of 14 participants with a mean ± SD of 24.05 ± 3.08 were invited on two additional days in order to examine the potential confounding effects of accommodative changes on changes in ocular biometry induced by cyan light. Among them, 6 were emmetropes (mean SER 0.00 ± 0.27 D) and 8 were myopes (mean SER − 3.09 ± 1.48). The participants were exposed to narrowband cyan light and broadband light using light-emitting glasses for 60 min on two separate days. For each light condition, the right eye was subjected to hyperopic defocus and left eye experienced no defocus. Ocular measurements were obtained 5 min after the introduction of defocus (before light exposure) and 60 min after light exposure (defocus + light exposure) in both eyes. Objective measurements of accommodation were performed using an open-field autorefractor (Shin-Nippon NVision-K 5001 autorefractor, Rexxam Co., Ltd.)^66^ under mesopic illumination of ~ 50 lx while participants were viewing a Maltese cross at 4 m (distance 0.28 × 0.25 m) and 30 cm (near 0.10 × 0.09 m) projected on a television and mobile phone, respectively.

The baseline measurement was taken unaided for emmetropic participants or with their habitual correction in participants with myopia. After the baseline measurement, participants were fitted with defocus CLs in the right eye and allowed 5 min of adaptation with the CLs. Following the adaptation period, a second autorefractor measurement was taken at both distance and near with the CLs. Participants were then instructed to wear the light glasses, and the room lights were dimmed to < 10 lx to minimise exposure to other wavelengths. Participants viewed a greyscale television placed 4 m away for the remaining duration of the 60-minute light exposure. Immediately after the 60 min of light exposure, participants were asked to remove the light glasses, and a third autorefractor measurement was performed. Once the third measurement was completed, the investigator removed the CLs. The mean sphero-cylindrical refraction of the eyes was calculated from a total of five readings at each session for the participants and the values were converted to vector notation of M (SE), J0, and J45. The mean change in M was used as a measure of best sphere refraction^58^.

### Statistical analysis

Following the data collection, the average of all ocular biometry and objective refraction data for each participant at each measurement session was calculated. The defocused (right eye) and non-defocused eye (left eye) were analysed separately in order to examine the influence of defocus in the right eye and any crossover effects in the left eye.

**Fig. 6:**
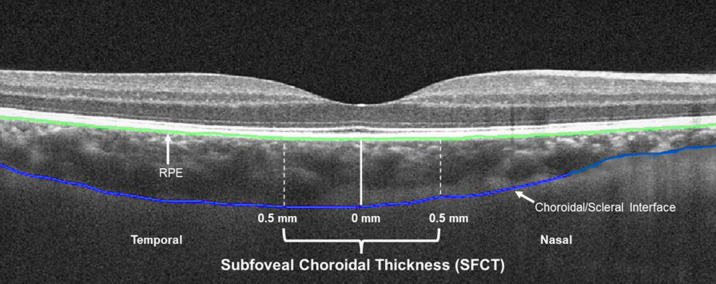
Example of an optical coherence tomography image from one measurement session, illustrating subfoveal choroidal thickness (SFCT) measured across the central 1 mm subfoveal region with the foveal pit as the reference point. SFCT was measured from the retinal pigment epithelium (RPE) to the inner edge of the choroid/scleral interface.

The OCT scans were exported from the instrument for further analysed using custom written software. The software automatically registered and performed chorio-retinal segmentation of the B-scan to calculate choroidal thickness defined as the axial distance between the retinal pigment epithelium and the inner edge of the choroid/scleral interface (Fig. 6). Automatic segmentations were checked for accuracy, and if required, were manually adjusted for any segmentation errors by an independent researcher who was blinded to the light conditions and the order of measurements. The SFCT was measured across the central 1 mm subfoveal region with the foveal pit as the reference point^58^ (Fig. 6). The segmented B-scan images featured a vertical red reference line that was automatically centred on the foveal pit. For scans where the reference line was not aligned with the centre of the foveal pit, it was manually repositioned at the foveal pit to accurately capture 0.5 mm on either side.

Statistical analyses were performed using IBM SPSS Statistics for Windows (version 26.0, ibm.com/au-en). A p-value below 0.05 was deemed statistically significant. All data were expressed as mean ± standard mean error (SEM). To examine the effect of cyan and broadband light on ocular biometry parameters between defocused and non-defocused eyes across time, a three-way repeated measures analysis of variance (ANOVA) was performed with “time”, “defocus” and “wavelength” as within-subjects factors and Holm–Sidak post-hoc tests for statistical significance. In an additional exploratory analysis, the effect of the two light conditions on AL and SFCT changes in myopes and emmetropes were analysed with three-way ANOVA, using ‘refractive error’ as between-subjects factor and ‘defocus’ and ‘wavelength’ as within-subjects variable. The change in accommodative response before (defocus only) and after 60 minutes of light exposure (defocus + light exposure) was calculated in both defocused and non-defocused eyes. To evaluate the within-session variability of each measured parameters on each measurement day, the average within-session SD and COV were calculated for each variable.

## Supplementary Information

Below is the link to the electronic supplementary material.

Supplementary Material 1

## Data Availability

The datasets generated and/or analysed during the current study are available from the corresponding author on reasonable request.
