# Supplementary material for: Short-term exposure to cyan light attenuates myopigenic effects of hyperopic defocus on ocular biometry in humans
Source: Sci Rep. 2026 Jan 9;16:4909. doi: 10.1038/s41598-026-35377-w (PMC12873225; doi:10.1038/s41598-026-35377-w)
Supplement: Supplementary file 1 — Supplementary Material 1 [file 41598_2026_35377_MOESM1_ESM.docx]

**Supplementary Table 1:** Overview of the mean within-subject variability for the repeated measures collected at each measurement session across different measurement days for axial length (AL), central corneal thickness (CCT), anterior chamber depth (ACD), lens thickness (LT), vitreous chamber depth (VCD), and subfoveal choroidal thickness (SFCT).

| **Measured variables** | **Mean within- subject standard deviation (mm)** | **Mean coefficient of variation (%)** |
| --- | --- | --- |
| AL | 0.009 | 0.04 |
| CCT | 0.005 | 0.49 |
| ACD | 0.016 | 0.56 |
| LT | 0.026 | 0.72 |
| VCD | 0.028 | 0.77 |
| SFCT | 0.011 | 3.74 |
